# Emerging trends and focus of research on the relationship between traumatic brain injury and gut microbiota: a visualized study

**DOI:** 10.3389/fmicb.2023.1278438

**Published:** 2023-11-03

**Authors:** Qiujing Du, Qijie Li, Guangneng Liao, Jiafei Li, Peiling Ye, Qi Zhang, Xiaotong Gong, Jiaju Yang, Ka Li

**Affiliations:** ^1^West China Hospital, Sichuan University/ West China School of Nursing, Sichuan University, Chengdu, China; ^2^Animal Experiment Center, West China Hospital, Sichuan University, Chengdu, China

**Keywords:** traumatic brain injury, gut microbiota, bibliometric, visualized study, emerging trends, research focus, Scopus

## Abstract

**Background:**

Traumatic brain injury (TBI) is one of the most serious types of trauma and imposes a heavy social and economic burden on healthcare systems worldwide. The development of emerging biotechnologies is uncovering the relationship between TBI and gut flora, and gut flora as a potential intervention target is of increasing interest to researchers. Nevertheless, there is a paucity of research employing bibliometric methodologies to scrutinize the interrelation between these two. Therefore, this study visualized the relationship between TBI and gut flora based on bibliometric methods to reveal research trends and hotspots in the field. The ultimate objective is to catalyze progress in the preclinical and clinical evolution of strategies for treating and managing TBI.

**Methods:**

Terms related to TBI and gut microbiota were combined to search the Scopus database for relevant documents from inception to February 2023. Visual analysis was performed using CiteSpace and VOSviewer.

**Results:**

From September 1972 to February 2023, 2,957 documents published from 98 countries or regions were analyzed. The number of published studies on the relationship between TBI and gut flora has risen exponentially, with the United States, China, and the United Kingdom being representative of countries publishing in related fields. Research has formed strong collaborations around highly productive authors, but there is a relative lack of international cooperation. Research in this area is mainly published in high-impact journals in the field of neurology. The “intestinal microbiota and its metabolites,” “interventions,” “mechanism of action” and “other diseases associated with traumatic brain injury” are the most promising and valuable research sites. Targeting the gut flora to elucidate the mechanisms for the development of the course of TBI and to develop precisely targeted interventions and clinical management of TBI comorbidities are of great significant research direction and of interest to researchers.

**Conclusion:**

The findings suggest that close attention should be paid to the relationship between gut microbiota and TBI, especially the interaction, potential mechanisms, development of emerging interventions, and treatment of TBI comorbidities. Further investigation is needed to understand the causal relationship between gut flora and TBI and its specific mechanisms, especially the “brain-gut microbial axis.”

## 1. Introduction

Traumatic brain injury (TBI) denotes a physical injury that affects the brain’s organic tissue resulting from an external force applied to the head either directly or indirectly (Kowalski et al., 2021). More than 50 million cases of TBI are reported globally each year, and it is the primary cause of death and disability in adults, with a disability rate of 43% and a mortality rate of 40% (Albert-Weissenberger et al., 2014; Hanscom et al., 2021). The significant social and economic burden imposed by TBI has garnered significant public attention. Nevertheless, despite substantial research over several decades, clinical progress in treating TBI has been limited due to the complex and heterogeneous nature of the injury (Hanscom et al., 2021). The development of new potential targets for the treatment and management of TBI is imminent.

The human gut is home to an estimated 10^13^ to 10^14^ microorganisms, which is approximately 10 times greater than the total number of human cells present in the body (Zhao and Zhang, 2017). Prior studies have unequivocally established the crucial role played by the gut microbiota in the preservation of health and the pathogenesis of diseases (Zhang et al., 2015). Notably, increasing preclinical and clinical evidence suggests that the intestinal microbiota might be a significant susceptibility factor for the onset of central nervous system diseases, and the “microbial-gut-brain” axis is an important mediating pathway (Ma Y. et al., 2019). Recent studies have provided compelling evidence demonstrating that TBI induces disturbances in intestinal microecology. Importantly, the restoration of a balanced intestinal microecology has emerged as a critical positive factor associated with improved prognostic outcomes following TBI (Rice et al., 2019). Treangen et al. (2018) demonstrated that following TBI, there was a significant reduction in the abundance of beneficial bacteria, including *Lactobacillus gasseri* and *Eubacterium ventriosum*, while the abundance of detrimental bacteria, such as *Eubacterium sulci*, exhibited an observable increase within a 24-h timeframe (Treangen et al., 2018). Moreover, after TBI, there is evidence to suggest that the levels and metabolic pathways of specific metabolites derived from the intestinal flora, such as short-chain fatty acids, glutamate, and tryptophan, undergo significant changes. These alterations have been observed to subsequently influence physiological effects associated with inflammation, the immune response, and neuroendocrine regulation, which may be associated with prognosis after TBI (Marć et al., 2022). It is noteworthy that emerging interventions targeting the gut flora are emerging as important entry points for the therapeutic management of TBI. A comprehensive meta-analysis revealed that prolonged probiotic supplementation exceeding a duration of 21 days effectively lowers levels of the inflammatory marker C-reactive protein (CRP) among patients diagnosed with TBI (Noshadi et al., 2022). Fecal transplantation, an emerging nonpharmacological intervention, aims to restore normal intestinal flora homeostasis and has shown remarkable results in treating TBI patients, including gastrointestinal and neurological benefits (Davis et al., 2022a,b). Simultaneously, the mechanism of interaction between TBI and gut flora has also attracted widespread interest, and the “microbial-gut-brain” axis targeting gut flora has been widely investigated as a targeted regulatory pathway for the treatment of TBI (Weaver, 2021). Subsequently, neuropsychiatric and metabolic-related disorders based on a series of pathophysiological changes in the “microbial-gut-brain” axis after TBI are becoming a focus of attention, as they affect the long-term health quality of life and prognosis of patients (Wang et al., 2021; Chiu and Anderton, 2023).

Given the promising clinical and scientific value of intestinal flora in the treatment and management of TBI, it is necessary to clarify the current trends and hotspots of research on the relationship between the two, especially in the areas of “intestinal microbiota and its metabolites,” “interventions,” “mechanism of action” and “other diseases associated with TBI.” Bibliometric analysis is a crucial tool for assessing research activity and trends and identifying future research directions. Knowledge maps visually capture and display complex information connections, providing an accurate representation of diverse pieces of information (Chen, 2004). The aim of this study is to reveal the emerging trends and focus of research on the relationship between traumatic brain injury and gut microbiota based on a visual approach of bibliometric analysis, with the aim of fostering advancements in the clinical development of TBI treatment and management.

## 2. Materials and methods

We downloaded relevant documents from the Scopus database, which is one of the most commonly used databases for high-quality bibliometric analysis and is widely accepted by researchers (Falagas et al., 2008; Mishra et al., 2021).

These term combinations were searched in the title, abstract, or keywords during this period: Terms related to TBI, i.e., ‘traumatic brain injury’ OR ‘cerebral injury’ OR ‘brain damage’ OR ‘brain injury,’ and terms related to the gut microbiota, i.e., (‘gut’ OR ‘intestin*’ OR ‘gastrointestin*’) AND (‘microbio*’ OR ‘microflora’ OR ‘flora’ OR ‘bacteri*’ OR ‘dysbiosis’ OR ‘microecology’ OR ‘16Sr*’ OR ‘metagenome’). There were no restrictions on language. The above search terms were extracted from the list of medical subject words provided by the National Library of Medicine or PubMed. Withdrawal of publications, editorials, notes, conference papers, book reviews or chapters, short surveys, letters, and corrections were excluded.

To analyze and visualize the bibliometric data in this study, CiteSpace and VOSviewer were used. CiteSpace is a diversified, time-sharing, and dynamic software for analyzing citations that was developed by Drexel University professor Chaomei Chen (Cheng et al., 2022). Its working principle is mainly based on the idea of “co-occurrence clustering.” First, the information units (such as references, keywords, subject words, authors, institutions, etc.) in the scientific literature are extracted, and then the network structure of different meanings is formed by reconstructing according to the connection type and strength between the information units. Finally, the measurement, statistical analysis and visualization of nodes, connections and network structure are carried out to discover the hidden patterns and laws of the knowledge structure of specific disciplines and fields (Cheng, 2020). In this study, we used CiteSpace 6.1. R6 to complete the following work: Develop the map of country and author co-occurrences; perform the knowledge map of journal, citation, and reference co-citation analyses; and create the map of keyword co-occurrences, and cluster knowledge maps, timeline views of keywords, and burst term maps are also created. VOSviewer is also a classic bibliometric analysis software (Ageel, 2022). Based on its unique advantages, we used its version of 1.6.18 to perform an “overlay visualization” reanalysis of high-frequency keywords.

## 3. Results

### 3.1. Time-trend distribution of documents

**Figure 1:**
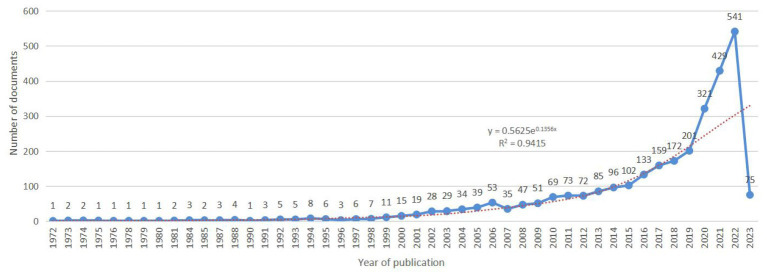
Time-trend distribution of documents in the field of the relationship between traumatic brain injury and gut microbiota.

We analyzed 2,957 documents published between September 1972 and February 2023 in total. An exponential growth trend was observed in the number of annual documents during this period (*y* = 0.5625e^0.1356x^, *R*^2^ = 0.9415). Figure 1 shows the specific number of annual documents.

### 3.2. Network analysis of scientific collaborations

**Figure 2:**
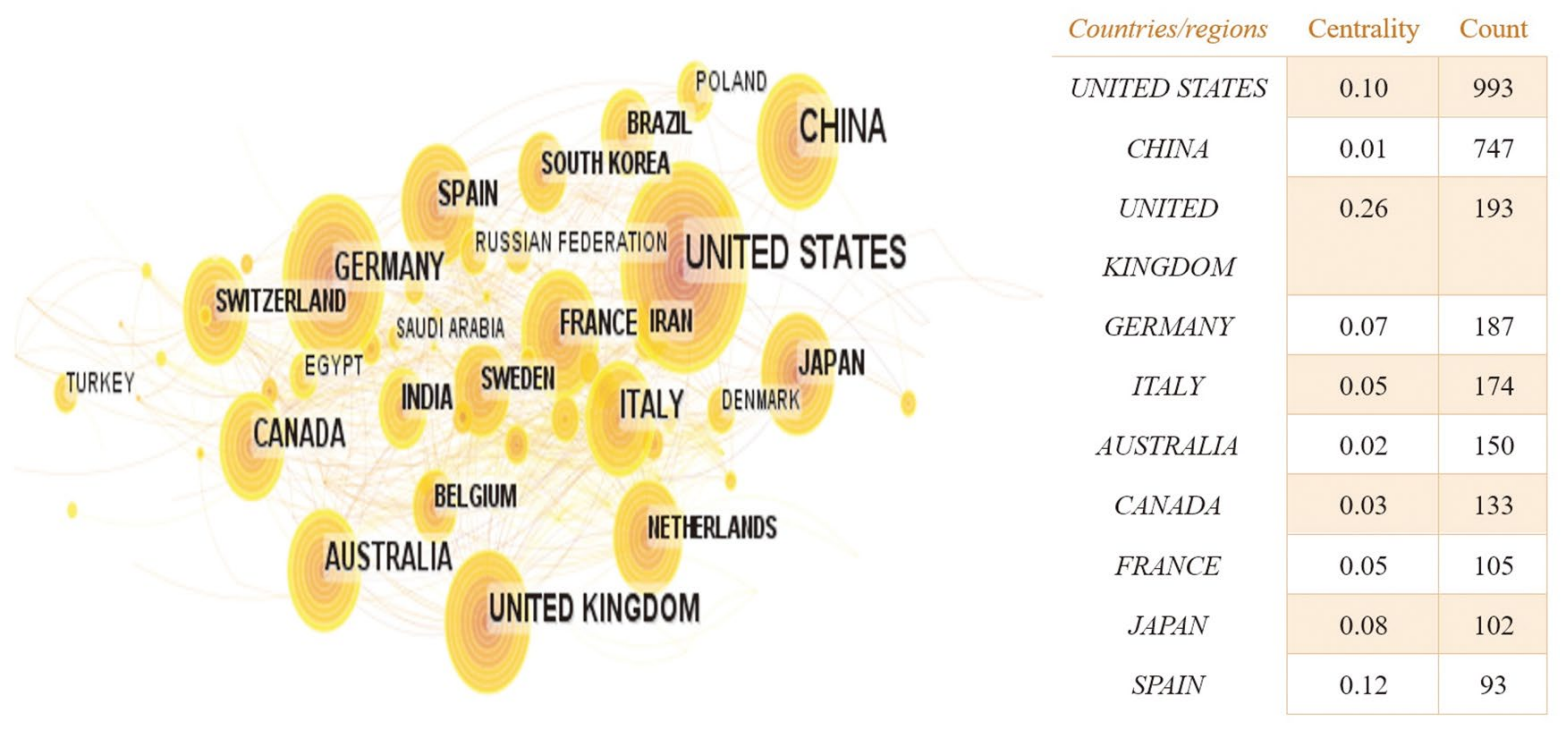
The country co-occurrence knowledge map of the relationship between traumatic brain injury and gut microbiota. Each node represents a country, and the size of the node is proportional to the number of documents published, the larger the node, the more documents published. Lines represent cooperative relations.

**Figure 3:**
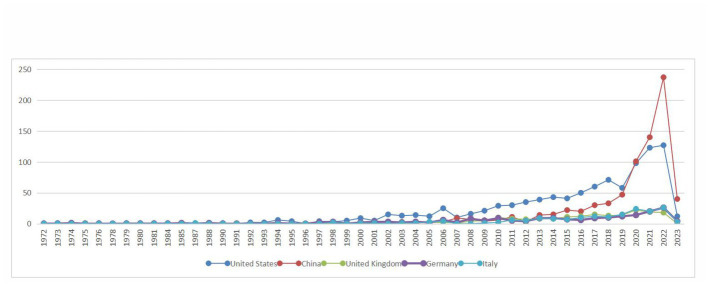
Annual distribution trends in the top five most published countries in the field of the relationship between traumatic brain injury and gut microbiota.

Based on the knowledge map of country co-occurrences (Figure 2), research teams from 98 countries or regions have published 2,957 documents on the relationship between TBI and intestinal microbiota. There are 105 nodes and 639 lines. Among the landmark nodes are the United States with 993, China with 747, the United Kingdom with 193, Germany with 187, and Italy with 174 texts. The lines in the map represent cooperative relations, and a close cooperative relationship has been formed between countries in their entirety. Centrality is a fundamental concept in network theory and graph theory that measures the importance or prominence of nodes (individual entities) within a network (a collection of interconnected nodes), and it helps us identify key nodes that play crucial roles in the network. In bibliometrics, degree centrality is one of the simplest centrality measures and is based on the number of direct connections (edges) a node has in the network. Its calculation formula is “Degree Centrality (C_d) = Number of Edges Connected to Node / (Total Number of Nodes – 1).” It measures how well-connected a node is to other nodes. Large centrality means that the relevant research has great influence, and highly influential countries include the United Kingdom, Spain, and the United States (centrality ≥0.1). In Figure 3, the annual distribution trends for the five most-published countries are shown. As of 2019, the United States has been in the leading position in the annual volume of documents published, but after that, China’s volume of documents published has rapidly increased to exceed that of the United States and become the leading country in the volume of documents published.

**Figure 4:**
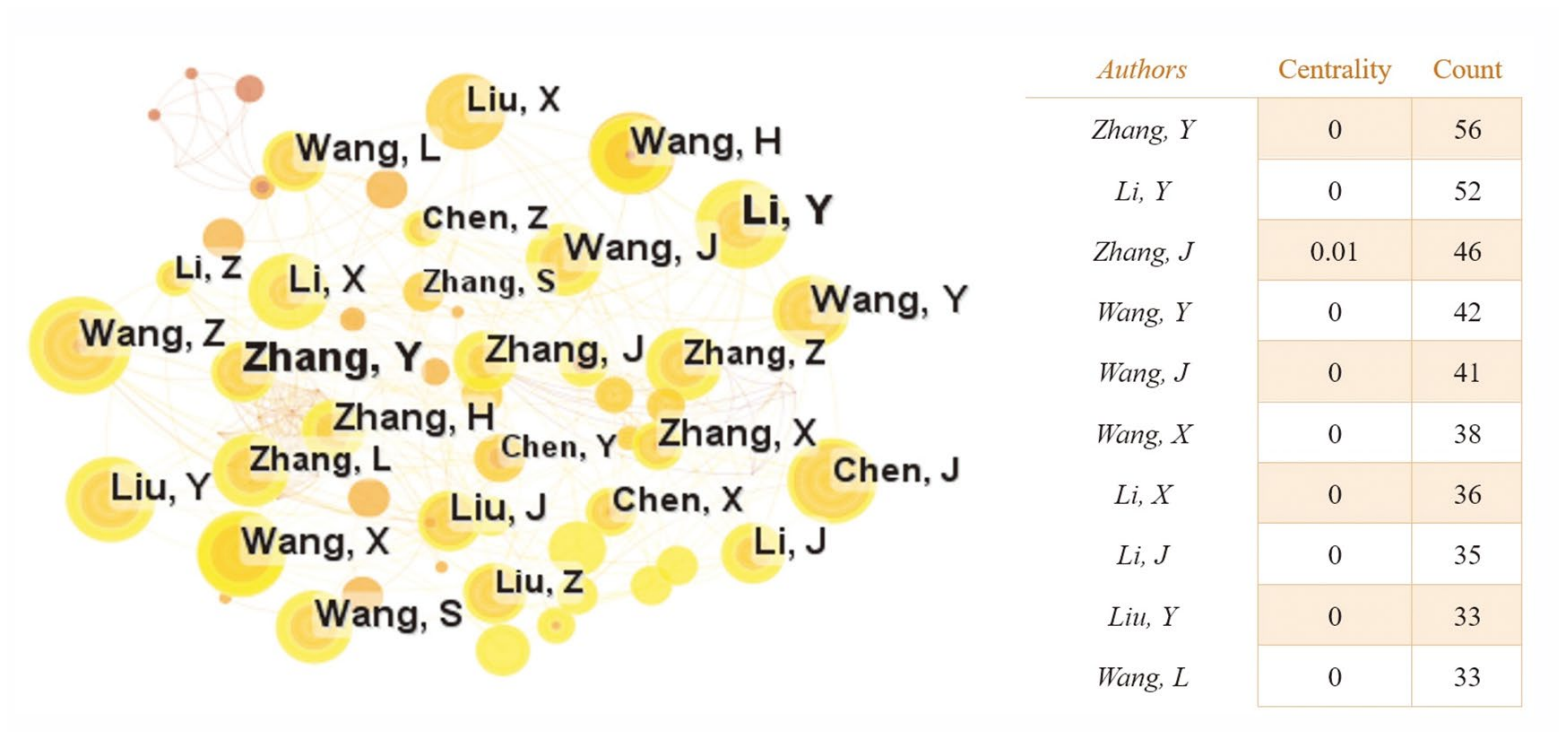
The author’s co-occurrence knowledge map of the relationship between traumatic brain injury and gut microbiota. Each node represents an author. The size of the node is in direct proportion to the number of papers published by the author. The larger the node, the more papers are published. Lines mean cooperation. The thickness of lines is proportional to the intensity of cooperation. The thicker the line, the greater the intensity of cooperation.

Based on the knowledge map of the author’s co-occurrences (Figure 4), there are 564 nodes and 1,387 lines, and the overall centrality is very low. Among the landmark nodes are Zhang Y, Li Y, Zhang J, Wang Y, and Wang J. Li Y, Wang Y, etc., have strong cooperation. Overall, close cooperative relationships have been formed among high-yield authors. The author’s cooperation years mainly focus on 2012–2023.

### 3.3. Analyses of citations and journals

In total, 160 journals published 2,957 documents on the relationship between TBI and the intestinal microbiota. As shown in Table 1, the top 10 journals publishing documents on the relationship between TBI and intestinal microbiota were analyzed. Among the top 10 journals, there are six professional journals in the field of neurology, one in the field of gastrointestinal science, one in the field of general medicine, and the others in the field of molecular science and immunology. The overwhelming majority of these journals exhibit a commendable standard, with a predominant representation from European and American publications.

**Table 1:** Top 10 journals that published documents on the relationship between traumatic brain injury and intestinal microbiota.

Journal	Frequency	JCR	IF	Country	Main ideas
International Journal of Molecular Sciences	66	Q1	6.208	United States	Strong emphasis on molecular biology and molecular medicine, including breakthrough experimental technical progress, fundamental theoretical problems et al.
Frontiers in Immunology	60	Q1	8.787	Switzerland	Relevant fields of immunology include the mechanism of immune system development and function, immunotherapy and regulation, inflammation, microbial immunology, nutritional immunology, bioinformatics, etc.
Journal of Neuroinflammation	34	Q1	9.589	England	Focuses on interactions of the immune system with the nervous system. This includes the roles of CNS immune mediators (such as microglia and astrocytes, and their expressed cytokines and chemokines) as well as the roles of peripheral neuro-immune interactions.
Journal of Neurotrauma	34	Q2	4.869	United States	To report on the latest advances in the clinical and laboratory investigation of traumatic brain injury and spinal cord injury, focusing on the basic pathobiology of central nervous system injury, while considering preclinical and clinical trials aimed at improving both the early management and the long-term care and recovery of traumatically injured patients.
Molecular Neurobiology	34	Q2	5.686	United States	Progress at the forefront of molecular brain research today.
Frontiers in Neurology	32	Q2	4.086	Switzerland	Focus on clinical neurology, neurochemistry and neurobiology
PLoS One	31	Q2	3.752	United States	Research in all fields and therapeutic areas of medicine
Brain Research	28	Q3	3.61	Netherlands	Neuroscience community that range in scope from issues in fundamental neurobiology to translational and clinical neuroscience.
World Journal of Gastroenterology	11	Q2	5.374	Peoples R China	Preclinical and clinical research related to gastrointestinal tract.
Progress in Neurobiology	10	Q1	10.885	England	Contributions from the broad field of neuroscience that apply neurophysiological, biochemical, molecular biological, and clinical neuroscience et al.

**Figure 5:**
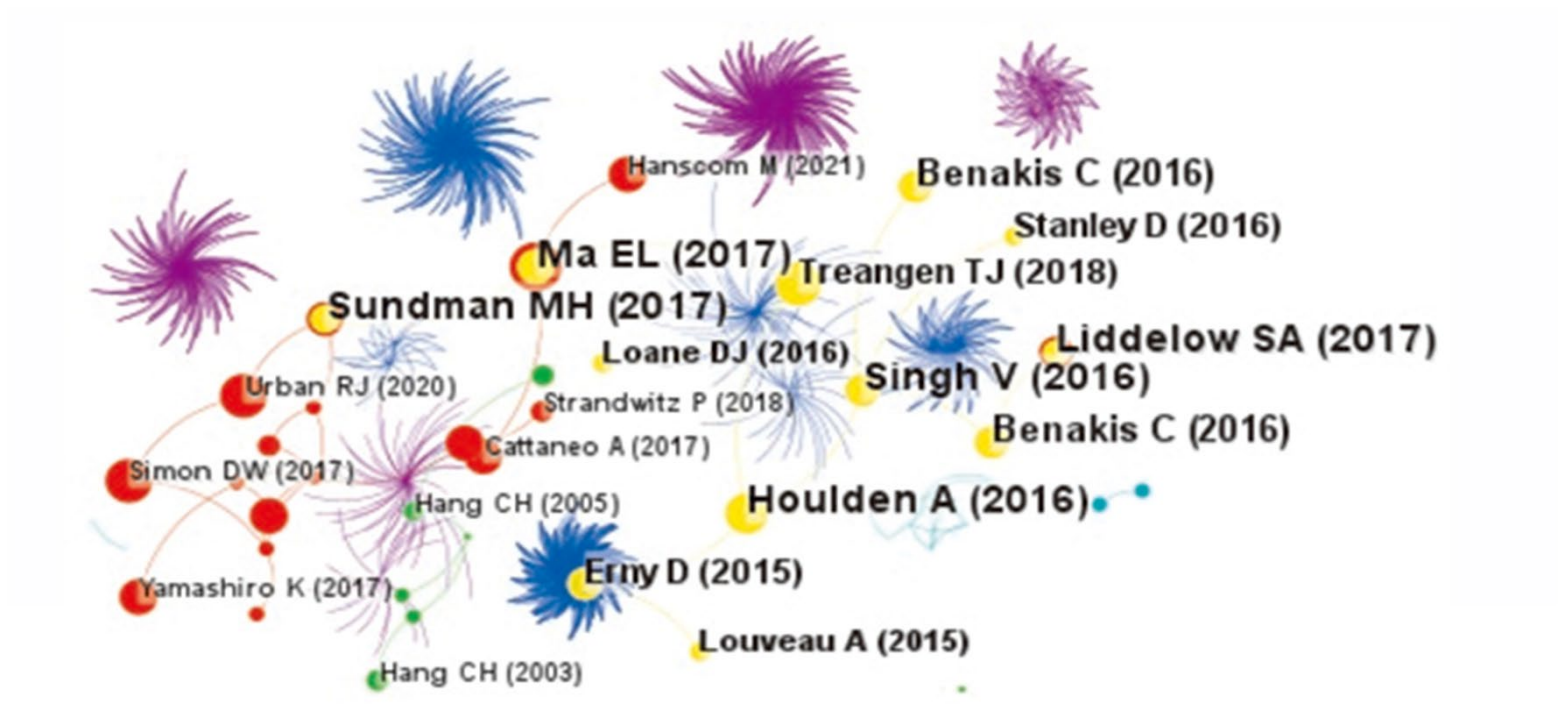
The co-citation knowledge map of the relationship between traumatic brain injury and gut microbiota. The size of the node is proportional to the number of co-citations. The larger the node is, the more the number of co-citations is. Lines represent the co-citation relationship. The change of cold and warm colors in different areas reflects the change in time dimension of references.

The reference co-citation knowledge map of the relationship between TBI and intestinal microbiota is shown in Figure 5. There are 837 nodes and 2,388 lines. The network of co-cited references is clear, and the high co-cited references are mainly concentrated in the warm color area, that is, relatively recent years. Table 2 shows the specific information of the top five co-cited references, and these highly co-cited references have been published in some top journals, such as Nature, Nat Neurosci, etc., mainly focusing on the brain-gut axis, glial cells, neuroinflammation, and microbiota.

**Table 2:** The top 5 co-cited references of the relationship between traumatic brain injury and intestinal microbiota.

First author	Year	Title	Journal	IF (2021)	Terms	Experimental techniques
Ma EL	2017	Bidirectional brain-gut interactions and chronic pathological changes after traumatic brain injury in mice.	Brain Behav Immun	19.227	Brain-gut axis, *Citrobacter rodentium*, Enteric glial cells, Traumatic brain injury, Neuroinflammation	GAPDH, 18S RNA
Sundman MH	2017	The bidirectional gut-brain-microbiota axis as a potential nexus between traumatic brain injury, inflammation, and disease.	Brain Behav Immun	19.227	Chronic Traumatic Encephalopathy, Gut-brain axis, Microbiota, Neurodegenerative disease, Microglia	16S rRNA
Houlden A	2016	Brain injury induces specific changes in the caecal microbiota of mice via altered autonomic activity and mucoprotein production.	Brain Behav Immun	19.227	Cerebral ischemia, Microbiota, Mucoprotein, Noradrenalin, Inflammation	DGGE, 16S rRNA, and Illumia MiSeq
Liddelow SA	2017	Neurotoxic reactive astrocytes are induced by activated microglia.	Nature	69.504	Reactive astrocytes, microglia, neuroinflammatory, Nervous diseases, Nerve injury	qPCR
Erny D	2015	Host microbiota constantly control maturation and function of microglia in the CNS.	Nat Neurosci	28.771	Microbiota, microglia, central nervous system, short chain fatty acids, nerve injury	16S rRNA

### 3.4. The emerging trends and the focus of the research were derived from keyword analysis

**Figure 6:**
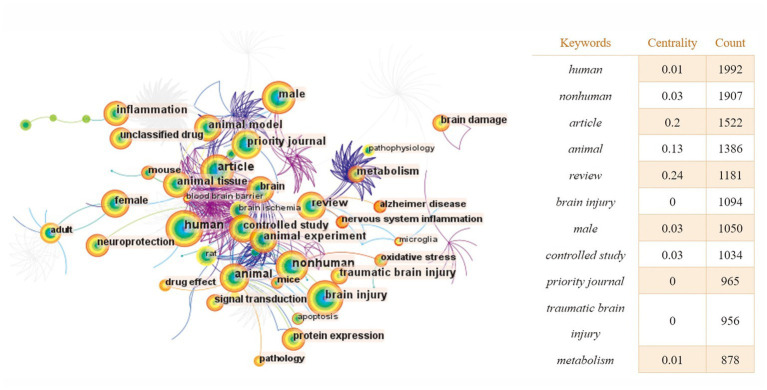
The keyword co-occurrence knowledge map of the relationship between traumatic brain injury and gut microbiota. Each node represents a keyword. The size of the node is proportional to the frequency. The larger the node, the higher the frequency. Lines represent a co-occurrence relationship. The color of the node is related to the occurrence time. The color from cold to warm indicates the time from far to near.

**Figure 7:**
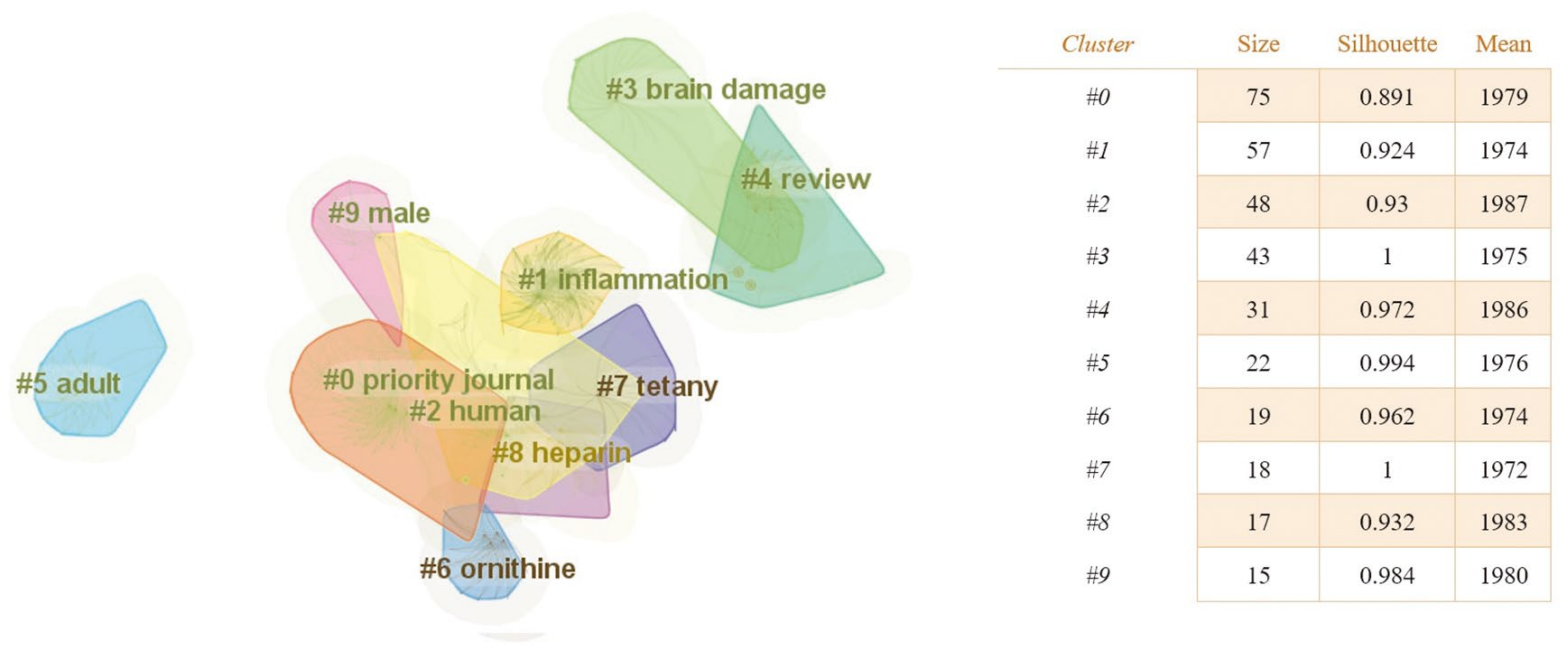
The keyword clustering knowledge map of the relationship between traumatic brain injury and gut microbiota. The clustering results are marked with # and obtained by the Log-likelihood rate (LLR) algorithm, while the clustering structure and legibility are mainly determined by two indicators: Modularity (Q value) and Silhouette (S value). The higher the *Q* value, the better the network clustering. When the *Q* value >0.3, it means that the cluster network structure is significant, and the *S* value is a measure of the homogeneity of the cluster map. When the *S* value >0.5, it means that the clustering result is reasonable.

**Figure 8:**
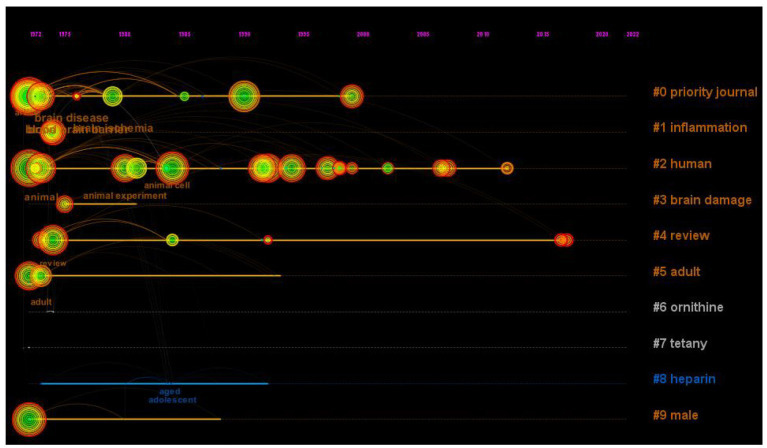
The timeline view of the relationship between traumatic brain injury and gut microbiota. In the timeline view, the keywords on the same horizontal line belong to the right cluster. The top is the time span.

**Figure 9:**
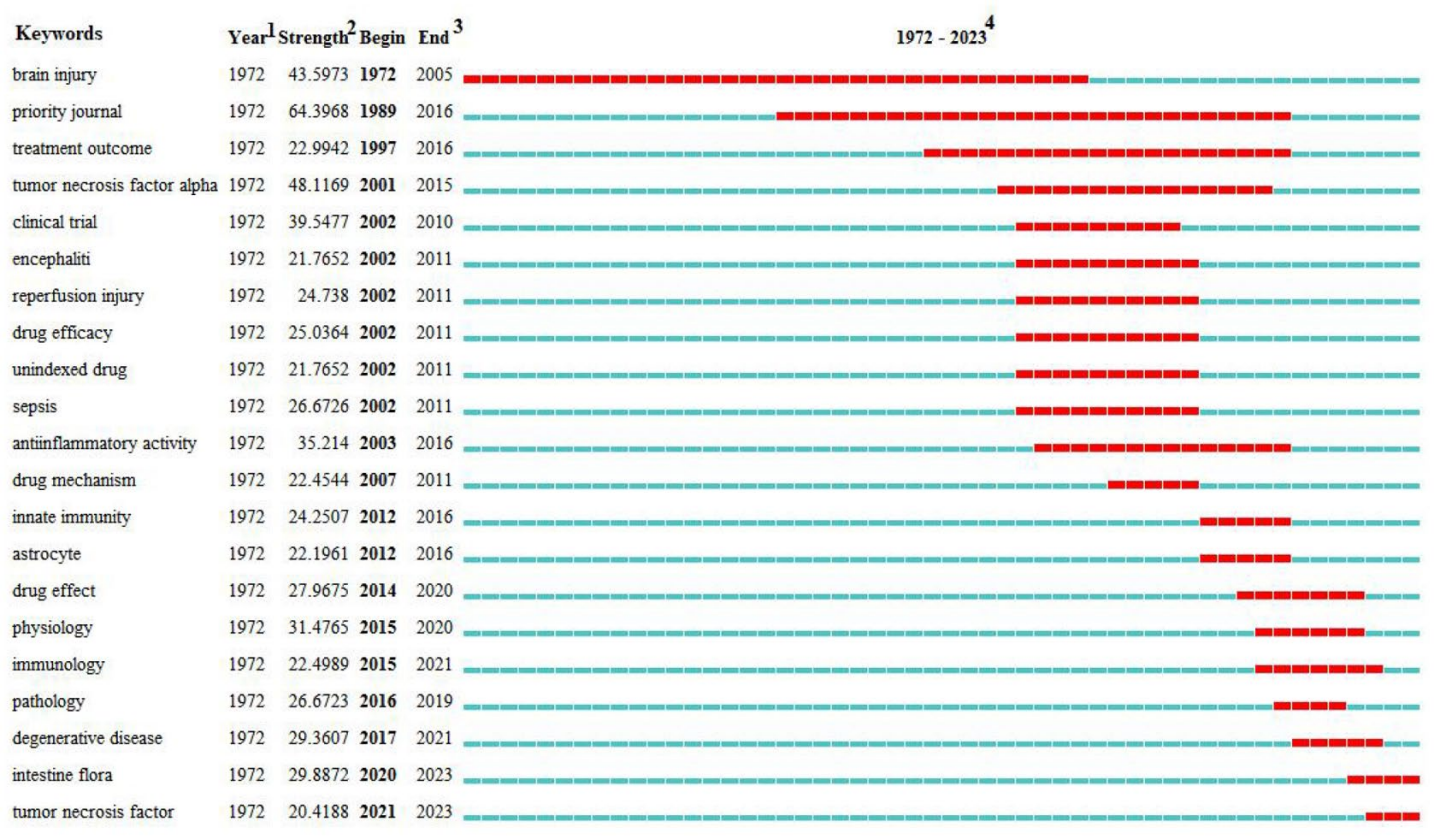
Top 21 keywords with the strongest citation bursts of the relationship between traumatic brain injury and gut microbiota. *^1^The year in which this keyword first appeared. ^2^The bursts’ strength of the keyword. ^3^The year in which this keyword begins and ends the burst. ^4^Red represents the period during which the keyword is burst.

**Figure 10:**
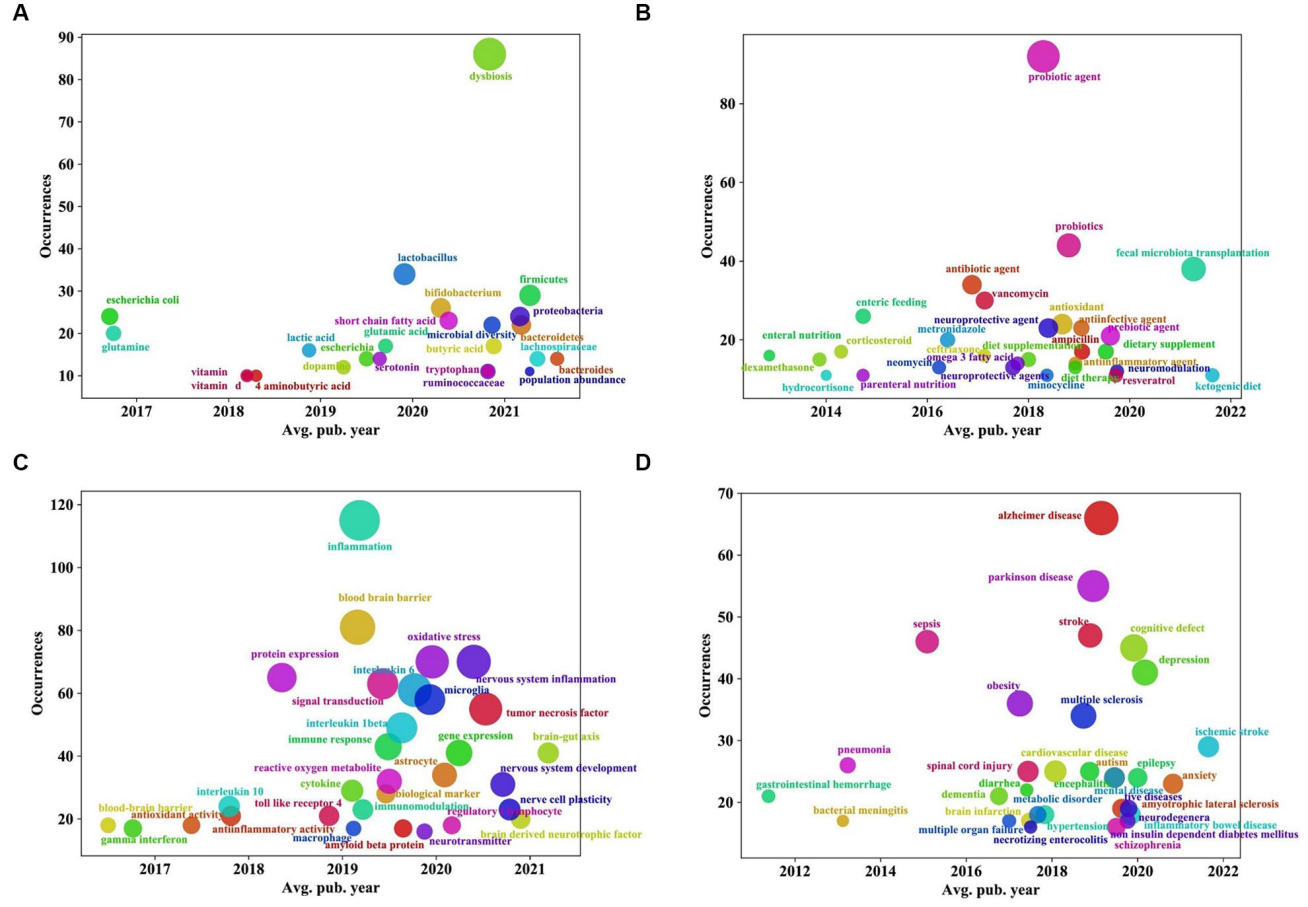
“Overlay visualization” analysis of keywords of the relationship between traumatic brain injury and gut microbiota. **(A)** Coordinate diagram of frequency occurrence of intestinal microbiota and its metabolites. **(B)** Coordinate diagram of frequency occurrence of interventions. **(C)** Coordinate diagram of frequency occurrence of mechanism of action. **(D)** Coordinate diagram of frequency occurrence of other diseases associated with traumatic brain injury. The abscissa represents the score calculated by the VOSviewer software based on the average year of publication, and the ordinate represents the weight of the keyword, that is, the frequency of occurrence.

With the help of CiteSpace software, we obtained a map of keyword co-occurrences (Figure 6), a map of clusters of keywords (Figure 7), a timeline chart of keywords (Figure 8), and a map of keyword burst terms (Figure 9). And the keywords were analyzed again using the “overlay visualization” mode of VOSviewer software to better identify research hotspots and frontiers; specifically, we categorized the relatively high frequency occurring keywords according to “intestinal microbiota and its metabolites,” “interventions,” “mechanism of action” and “other diseases associated with traumatic brain injury” (Figures 10A–D).

There are 404 nodes and 1,247 lines in the map of keyword co-occurrence knowledge (Figure 6). Larger landmark nodes include human, nonhuman, article, animal, review, brain injury, and male, which represent the most important parts of the field of the relationship between TBI and gut microbiota. Turning points with more connections are brain, female, adult, and animal, suggesting that they have a higher centrality in the domain and were related to a larger number of keywords.

As shown in Figure 7, there were 10 clusters in the research area of the relationship between traumatic brain injury and gut microbiota. Additionally, Table 3 shows the top 5 most typical labels within each cluster.

**Table 3:** The most typical label in each cluster.

Cluster	Label
#0	Priority journal; brain; human; neuroprotection; pain.
#1	Inflammation; unclassified drug; protein interaction; middle aged; metabolic disorder.
#2	Human; review; animal experiment; animal tissue; animal model.
#3	Brain damage; clostridium perfringen; meningoencephalitis; lead; enterotoxemia.
#4	Review; Alzheimer disease; male; article; controlled study.
#5	Adult; female; middle aged; aged; major clinical study.
#6	Ornithine; prenatal diagnosis; electroencephalography; alkalosis; urea cycle.
#7	Tetany; megacolon; vomiting; intestine obstruction; intestinal atresia.
#8	Heparin; digestive system; convulsion; birth injury; differential lung ventilation.
#9	Male; review; Parkinson’s disease; Alzheimer’s disease; ammonia.

Research hot-spot evolution can be seen through the combination of the map of clusters of keywords (Figure 7), the map of the timeline chart of keywords (Figure 8), and the map of keyword burst terms (Figure 9). From 1972 to 1997, brain injury, inflammation, brain damage, adult, ornithine, tetany, heparin, and male were gradually attracting the attention of academic circles in relevant fields. Over the next 18 years (1998–2016), the focus gradually shifted to intervention effect, tumor necrosis factor-alpha, clinical trial, encephalitis, reperfusion injury, sepsis, anti-inflammatory activity, mechanism of action, innate immunity, and astrocytes. In recent years (2017–2023), physiology, immunology, pathology, degenerative disease, intestine flora, tumor necrosis factor, and effects of action have progressively emerged as the central focus of scholarly researchers.

Additionally, as shown in Figures 10A–D, in terms of “intestinal microbiota and its metabolites,” before 2019, the focus was mainly on *Escherichia coli* and glutamine. In recent years, in addition to some beneficial bacteria, such as *Lactobacillus* and *Bifidobacterium*, and other intestinal microflora, such as *Firmicutes*, *Proteobacteria*, *Bacteroidetes*, *Lachnospiraceae*, dysbiosis, and microbial diversity, an increasing number of metabolites or derivatives of intestinal microflora, such as short-chain fatty acids (SCFAs), glutamic acid, tryptophan, serotonin, etc., have received increasing attention (Figure 10A). Regarding “interventions,” the focus of research has gradually shifted from antibiotic interventions such as vancomycin, metronidazole, and neomycin, hormone interventions such as corticosteroids, dexamethasone, and hydrocortisone, and measures related to enteral and parenteral nutrition to probiotic/prebiotic interventions and the use of neuroprotective agents and antioxidants. Moreover, dietary therapies such as ketogenic diets (KD) and omega-3 fatty acids as well as fecal microbiota transplantation techniques have gradually become the focus of attention in recent years (Figure 10B). Regarding the “mechanism of action,” the focus in recent years has been on central and peripheral inflammatory reactions (mainly involving inflammatory markers such as interleukin-6, tumor necrosis factor, and interleukin-1 β etc.), oxidative stress reactions, and immunoregulation; in addition, the mechanisms related to the brain-gut axis, astrocytes, and microglia have also been a research hotspot in recent years (Figure 10C). For “the related diseases,” in recent years, in addition to some neurological diseases, such as Alzheimer’s disease, Parkinson’s disease, and stroke, psychiatric disorders, such as depression, anxiety, autism, and schizophrenia, and metabolic diseases, such as obesity, metabolic disorder, and noninsulin-dependent diabetes mellitus, have gradually attracted researchers’ interest (Figure 10D).

## 4. Discussion

### 4.1. Overall review of the study

From Figure 1, it can be seen that the annual publication volume of documents related to the relationship between TBI and intestinal microflora has increased exponentially, suggesting that this area is a research hotspot and that its level of attention will continue to increase. To discover more links between TBI and intestinal microflora, researchers need to keep an eye on trends in pertinent disciplines on an ongoing basis. The correlation between TBI and intestinal flora has been studied worldwide (Figure 2). The United States has the largest total number of publications, possibly related to the launch of the National Institute of Neurological Disorders and Stroke (NINDS) Traumatic Brain Injury (TBI) Program and Gut Microbiota Brain AXIS program (Nielson et al., 2017; Integrative HMP (iHMP) Research Network Consortium, 2019). Interestingly, the number of publications in China has increased rapidly since 2019, surpassing that of the United States, which may be related to the emphasis placed by the National Natural Science Foundation of China and China’s National Key R&D Plan on research related to the role of gut microbes in disease onset, development and regression (National Natural Science Foundation of China, 2022). In addition, the United Kingdom has the greatest influence in publishing research in related fields, which may be related to the fact that most of the relevant studies were published in internationally influential authoritative journals and that relevant research results were produced by internationally renowned laboratories (Houlden et al., 2016; Parker et al., 2020). It is recommended that while countries should focus on the quantity of research in related fields, they should also focus on the quality of research, such as innovation and the scientific nature of methods, to improve the international impact of related research. In recent years, authors in research-related fields have formed relatively close collaborations mainly with high-yielding authors, but collaborators are mainly from China, and international collaborations are less frequent. In the future, international collaboration should be strengthened to jointly advance scientific progress in the relationship between TBI and intestinal flora in a global scientific context.

When researchers publish and read documents related to the relationship between TBI and intestinal microbiota, the top 10 journals (Table 1) might be given primary consideration. Scholars in relevant fields could read the high-citation papers to discover the hotspots of research in the field. In addition, novices can read the highly co-cited papers (Table 2) to understand the connections and significant findings between studies in the field.

### 4.2. Overview of research trends and directions of increasing interest

As seen from the keyword-related knowledge maps (Figures 6–9), research related to the relationship between TBI and gut flora has focused on the following areas: “types of study subjects,” “intervention methods,” and “mechanisms.” Researchers have conducted numerous relevant studies on both humans and nonhumans. Studies related to the relationship between TBI and gut flora have been analyzed according to the different ages [e.g., young mice (Chen et al., 2022) and adult mice (Houlden et al., 2016), gender (male and female) (Luo et al., 2022)], and health/disease characteristics of the subjects, probably because previous studies have found differences in the composition of the gut flora of study subjects with different characteristics (Marathe et al., 2012; Sun et al., 2022), and their changes, related roles, and mechanisms in TBI with different characteristics might also differ. However, studies on the relationship between TBI and gut flora throughout the lifespan (infancy, adolescence, adulthood, menopause, and old age) are insufficient, and the relationship between TBI with different health/disease characteristics (e.g., TBI with neurodegenerative diseases) and gut flora also needs to be further explored. In addition, interventions including pharmacological treatments (Hu et al., 2023), nutritional support techniques (Curtis and Epstein, 2014), probiotic/prebiotic supplementation (Brenner et al., 2017), dietary therapies (Thau-Zuchman et al., 2021; Li et al., 2022), and fecal transplantation techniques (Du et al., 2021) have been used in studies related to this field. Researchers could choose different intervention methods according to their research objectives or develop new intervention methods based on the above methods to enrich the research in related fields and promote the safe, comfortable, and rapid recovery of TBI patients. As an important biomarker, the mechanism related to the relationship between the intestinal flora and TBI is also a focus and hot topic of research in this field (Ma et al., 2017). Inflammatory response (Xiao et al., 2022), oxidative stress (Ni et al., 2019), immune regulation (Seki et al., 2021), central nervous cell response (Zheng et al., 2022), and brain-gut axis (Zhanfeng et al., 2022) are the main keywords related to the current mechanism.

#### 4.2.1. Research trends and hot spots of “intestinal microbiota and its metabolites”

The keyword “overlay visualization” analysis using VOSviewer showed the changes in high-frequency keywords over time in the “intestinal microbiota and its metabolites,” “interventions,” “mechanism of action” and “other diseases associated with traumatic brain injury” categories.

Before 2019, *Escherichia coli* (*E. coli*) among the intestinal flora was a hot research topic in the field. In 1998, researchers used the *E. coli* β-galactosidase reporter gene to localize cholecystokinin transcripts in the brains of transgenic TBI mice (Itoh et al., 1998), and this was the first time *E. coli* entered the arena of TBI-related research as a primary subject of study. In the following 20 years, studies on the relationship between TBI and *E. coli* gradually increased, mainly focusing on the pathogenic mechanism of *E. coli* in TBI (Unzicker et al., 2011), the regulatory effect of interventions on *E. coli* in individuals with TBI (Watanabe et al., 2004), and the changes of *E. coli* after TBI (Patel et al., 2016). In addition, the physiological and clinical benefits of glutamine in TBI are of interest during this time (Falcão de Arruda and de Aguilar-Nascimento, 2004; Feng et al., 2007). After 2019, the focus of researchers gradually shifted to the modulatory effects of beneficial bacteria such as *Lactobacillus* and *Bifidobacterium* on TBI (Chen et al., 2022, 2023; Kim et al., 2022; chinazx.org.cn, 2023; Zhang et al., 2023) and the changes in the structure and diversity of the intestinal flora after different types of TBI (mild, moderate, severe) (Matharu et al., 2019; Brenner et al., 2020a; Yang et al., 2022). Importantly, in recent years, researchers have become increasingly aware that the physiological effects of intestinal flora are largely attributable to their metabolites or derivatives. And rather than bacterial species equilibrium, the metabolic status of the microbiota plays a more significant role (Marć et al., 2022), and as a result, numerous studies have been conducted on intestinal flora metabolites or derivatives, mainly including SCFAs, glutamate, tryptophan, and serotonin. SCFAs are organic monocarboxylic acids released after metabolic fermentation of indigestible dietary fiber, plant polysaccharides or amino acids and are important metabolites derived from the intestinal microbiota that play an important role in maintaining health (Morrison and Preston, 2016; Fock and Parnova, 2023). Recent studies have found that the significant decrease in fecal levels of SCFAs following TBI may be related to the dysbiosis of intestinal microecology (a decrease in the number of short-chain fatty acid-producing bacteria) caused by TBI (Medel-Matus et al., 2022). In addition, SCFAs depletion after TBI may be associated with dysregulation of the proinflammatory immune phenotype and the development of secondary brain damage (Opeyemi et al., 2021). Interestingly, there is a growing body of research supporting the neuroprotective role of SCFAs in TBI. Previous studies have reported that valproic acid has antiepileptic and neuroprotective effects in a mouse model of TBI (Bambakidis et al., 2016) and that sodium butyrate exerts neuroprotective effects by restoring the structure and function of the blood–brain barrier in mice with TBI (Li et al., 2016). A recent study found that supplementation with SCFAs can improve spatial learning ability in TBI mice (Opeyemi et al., 2021). And a latest study reviewed the relevant mechanisms of the neuroprotective effects of SCFAs, mainly involving the effects of SCFAs mediated by G-protein-coupled receptors, inhibition of histone deacetylases by SCFAs stimulates acetylation of histones and nonhistone proteins, and transcription factors NF-KB and Nrf2 interact with these signaling pathways (Fock and Parnova, 2023). However, the signaling pathways that mediate the role of SCFAs are still unclear, especially the details of the involvement of short-chain fatty acid receptors. In the future, further exploration of specific or new mechanisms for the neuroprotective effects of SCFAs is needed. Glutamate is a major excitatory neurotransmitter that can be produced by intestinal microorganisms and plays a key role in synaptic plasticity, learning, and memory (McGrath et al., 2022). In recent years, researchers have found that TBI might lead to the abnormal release of glutamate, leading to excitotoxic secondary nerve damage (Yilmaz et al., 2019). In addition, glutamate, as a marker of brain metabolic status, may have certain predictive and diagnostic value in the risk of neurodegeneration after TBI (Eisele et al., 2020). Researchers have also conducted more research on tryptophan and serotonin. It is found that early use of selective serotonin reuptake inhibitors can improve neurological function in patients with TBI (Zhang et al., 2021). However, some studies have shown that 5-HT, a metabolite of tryptophan, is neurotoxic, and its induced increase in brain serotonin levels can disrupt the blood–brain barrier and lead to vasogenic brain edema, which leads to adverse mental and behavioral changes after TBI (Sharma et al., 2019). Therefore, future research needs to further explore the exact role of tryptophan metabolism and serotonin neurons in TBI to promote neural rehabilitation in patients with TBI.

#### 4.2.2. Research trends and hot spots of “interventions”

In recent years, some emerging interventions, mainly nondrug-based interventions, have gradually become a research hotspot in this field. Probiotics have been the most discussed and researched interventions in recent years. Probiotics are living microorganisms that, when given in sufficient amounts, can have beneficial effects on host health (Brenner et al., 2020b). An increasing number of studies have found that the use of probiotics has benefits for TBI. For example, a clinical randomized controlled trial has shown that probiotic supplementation could reduce systemic inflammatory reactions and promote the rehabilitation of patients with severe TBI (Wan et al., 2020). Animal experiments have also shown that supplementing probiotics can alleviate inflammatory intestinal injury and enhance immune tolerance in TBI rats (Hou et al., 2021). In addition, the use of probiotics in patients with moderate to severe TBI is associated with reduced infection rates, the incidence of ventilator-associated pneumonia, improvement in gastrointestinal dysfunction, and shorter hospital stay in the intensive care unit (Aghakhani, 2022). Recently, researchers have gradually begun to focus on the neuropsychiatric benefits of probiotics for TBI. Some studies have found that supplementation with probiotics can improve neuronal defects and sensorimotor deficits in TBI mice (Ma Q. et al., 2019). However, the cognitive and behavioral effects of probiotic supplementation in TBI patients remain to be determined. Fecal bacteria transplantation is a method of transplanting fecal microbiota from healthy donors into diseased recipients, achieving therapeutic effects by restoring the microbial structure and diversity of diseased recipients (Kwak et al., 2020). Currently, this technology has received much attention, and studies have demonstrated that fecal bacteria transplantation could normalize the intestinal microecology of TBI mice and has neuroprotective effects (Davis et al., 2022a,b). However, how to establish the criteria for qualified donors and the safety and effectiveness of this technology in the TBI population need to be further confirmed. Recently, dietary therapy has received widespread attention from researchers due to its advantages of low cost, noninvasiveness, and high patient acceptance. Currently, most related studies are focused on KD and omega-3 fatty acids (Mao et al., 2021). It has been reported that the intestinal flora plays a significant role in the neuroprotective effect of KD on TBI. For example, the intestinal flora can affect brain excitability by regulating the peripheral metabolites of KD (Krakovski et al., 2022). The protective effect of omega-3 fatty acids on brain injury by altering the intestinal microbiota has also been reported (Lei et al., 2016). In addition, the use of neuroprotective agents and antioxidants has also been a research hotspot in recent years, but the role of the intestinal flora in their action process is still unknown.

#### 4.2.3. Research trends and hot spots of “mechanism of action”

In terms of the “mechanism of action,” the inflammatory response is the most widely studied mechanism, which may be due to it being the most critical factor influencing the pathological process of TBI and playing an important role in the process of secondary brain injury (Tschoe et al., 2020). How to regulate the inflammatory response after TBI is the key to the treatment and rehabilitation of TBI (Faden et al., 2016). Recently, the intestinal microflora as a potential target for regulating the inflammatory response after TBI has gradually aroused widespread interest among researchers. For example, there is evidence that changes in the structure and diversity of the intestinal microflora can regulate neuroinflammation after TBI by activating NLRP3 inflammatory bodies and releasing inflammatory factors, affecting the process of secondary brain injury (Zhang et al., 2022). In addition, there have also been a few reports on the regulatory effects of intestinal microflora on oxidative stress (Kumari et al., 2022) and immune responses (Celorrio et al., 2021, 2023) (mainly involving glial cell-related reactions) after TBI. However, currently, an emerging term has aroused heated attention in this field, namely, the “brain-gut axis”/“brain-gut-microbial axis.” This term summarizes the aforementioned mechanisms related to the inflammatory response, oxidative stress, and immune regulation of intestinal flora to TBI while emphasizing the two-way connection between microorganisms, gut, and brain. On the one hand, TBI can induce systemic inflammatory and immune responses, as well as dysfunction of the autonomic and intestinal nervous systems, leading to intestinal damage and intestinal microecological disorders, and intestinal dysfunction in turn causes intestinal bacteria to release lipopolysaccharide (LPS), which enters the circulation and reaches the brain, further exacerbating neuroinflammation (Hanscom et al., 2021). On the other hand, normalization of the intestinal microflora or supplementation with beneficial bacteria can reduce oxidative stress reactions and improve neuroinflammation in TBI through Toll-like receptor 4-dependent mechanisms (Weaver, 2021). In the future, the targeting mechanism, signal transduction, and further role of the intestinal flora in brain-gut communication need to be further explored to promote the development of new TBI treatment interventions.

#### 4.2.4. Research trends and hot spots of “other diseases associated with traumatic brain injury”

As far as “other diseases associated with traumatic brain injury” are concerned, neurological diseases, psychiatric disorders, and metabolic diseases have received much attention from researchers in recent years. A substantial body of evidence suggests that individuals with TBI are at greater risk of developing neurodegenerative disorders, including Alzheimer’s disease and Parkinson’s disease (Kokiko-Cochran and Godbout, 2018; Delic et al., 2020). Newer research findings indicate that alterations to the “microbial-gut-brain axis” resulting from TBI may play a significant role in the etiology of neurodegenerative disorders (Chiu and Anderton, 2023). Specifically, the TBI-induced biochemical cascade can disrupt gut microbiome homeostasis, leading to increased intestinal permeability and resulting in persistent, low-grade inflammation, exacerbating the peripheral effects of TBI, and such gastrointestinal changes may activate immune and inflammatory responses that, in turn, transduce to the central nervous system, initiating a cascade of events that ultimately promote neurodegeneration (Chiu and Anderton, 2023). Thus, the gut flora may be a potential target for elucidating the temporal relationship between TBI and the development of neurodegenerative diseases. Additionally, TBI is also considered one of the risk factors for stroke (Qu et al., 2022), and the imbalance of intestinal microbiota, as well as further immune dysfunction and upregulation of the inflammatory response caused by TBI, are considered possible mechanisms (Singh et al., 2016). The association between TBI and psychiatric disorders has been confirmed in many previous studies (Merritt et al., 2018; Yeh et al., 2020). Interestingly, more recent studies have shown that a history of childhood TBI was associated with the onset and severity of psychiatric disorders in adulthood (Arif et al., 2021), suggesting that the early years of a researcher’s life are an important window for the prevention and management of disorders and should be given focused attention. However, the exact mechanisms of the relationship are not fully clarified, and the dysregulation of gut ecology caused by TBI may contribute to the onset and development of psychiatric disorders through altered neurotransmitter levels, immune function, and inflammatory responses (Socała et al., 2021). The involvement of TBI in the development of metabolic diseases has been of increasing interest to researchers, with mechanisms involving disruption of intestinal homeostasis, dysregulation of bile acid metabolism, dysregulation of amino acid metabolism, and imbalance of the inflammatory response (Wang et al., 2021; You et al., 2022), but such studies have mainly focused on animal studies, and future studies in human populations are needed to guide the development of interventions for TBI-induced metabolic diseases with the intestinal flora as a target.

### 4.3. Strengths, limitations, and future directions

This study was based on bibliometrics using a visual approach to review the trends and priorities of research on the relationship between TBI and gut microbiota, with a detailed focus on the current status and trends of research in the areas of “intestinal microbiota and its metabolites,” “interventions,” “mechanism of action” and “other diseases associated with traumatic brain injury,” providing potential insights into the pathogenesis and mechanism of action of TBI, the development of targeted interventions, and the clinical management of TBI co-morbidities. Inevitably, however, there are some limitations to this study. First, it is pertinent to note that this study relied solely on documents sourced from the Scopus database. Despite the widespread recognition among scholars for the Scopus database as a reliable and comprehensive resource for conducting bibliometric analyses, it is crucial to acknowledge the potential for the inadvertent omission of certain studies pertaining to the association between gut microbiota and TBI, which merits careful consideration due to its potential influence on the study’s findings. Second, the time point of the literature included in this study is up to February 2023, and the trends and focus of the field beyond this time point were not included. In the future, the causal relationship between gut flora and TBI and the exact mechanism of gut flora metabolites in the development of TBI need to be further investigated to provide preclinical evidence on the use of gut flora in the treatment of TBI, which will also provide a basis for personalized and precise treatment of TBI using gut flora as a target. Additionally, the efficacy and mechanism of action of nonpharmacological interventions such as probiotics in the treatment of TBI, especially in neuropsychiatric aspects, need to be further explored. Importantly, the investigation of the regulatory function of the “brain-gut-microbial axis” concerning TBI also constitutes a significant area of research for future exploration. Also, the specific mechanisms of gut flora as a potential target for elucidating the temporal relationship between TBI and the development of neuropsychiatric and metabolic disorders need to be further explored. Finally, the establishment of uniform procedures for the production of enteric microbiological preparations, together with the development of more practical and effective techniques for the detection of microflora, are also necessary to form the basis for the development of related industries.

## 5. Conclusion

This study performed a visual bibliometric analysis of studies related to the relationship between TBI and gut microbiota included in the Scopus database from September 1972 to February 2023. The relationship between TBI and gut microbiota has attracted increasing interest from researchers in recent years, with the number of relevant documents increasing each year. Although the topic has been researched worldwide, there is some variation in the number and impact of studies in different countries and regions, and international cooperation is relatively lacking. Important research themes in this area focus on “types of study subjects,” “intervention methods,” and “mechanisms.” “Intestinal microbiota and its metabolites,” “interventions,” “mechanism of action” and “other diseases associated with traumatic brain injury” are the four potential and valuable research points. In the future, the causal relationship between gut flora and TBI and the specific mechanisms involved, especially the “brain-gut-microbial axis,” remain to be further elucidated. Targeting gut flora, the development of precisely targeted interventions, the clinical management of TBI co-morbidities, and the development of microbial precision detection technologies are also important research directions in prospective periods.

## Author contributions

QD: Data curation, Formal analysis, Investigation, Supervision, Writing – original draft. QL: Data curation, Formal analysis, Investigation, Supervision, Writing – original draft. GL: Data curation, Writing – original draft. JL: Data curation, Writing – original draft. PY: Data curation, Writing – original draft. QZ: Data curation, Writing – original draft. XG: Data curation, Writing – original draft. JY: Data curation, Writing – original draft. KL: Conceptualization, Funding acquisition, Project administration, Supervision, Validation, Writing – review & editing.
